# Data Leakage in Deep Learning for Alzheimer’s Disease Diagnosis: A Scoping Review of Methodological Rigor and Performance Inflation

**DOI:** 10.3390/diagnostics15182348

**Published:** 2025-09-16

**Authors:** Vanessa M. Young, Samantha Gates, Layla Y. Garcia, Arash Salardini

**Affiliations:** 1 Glenn Biggs Institute for Alzheimer’s and Neurodegenerative Diseases, University of Texas Health Science at San Antonio, San Antonio, TX 78229, USA; youngv1@uthscsa.edu (V.M.Y.); gatess2@uthscsa.edu (S.G.);; 2 Graduate School of Biomedical Sciences, University of Texas Health Science at San Antonio, San Antonio, TX 78229, USA; 3 Department of Neurology, University of Texas Health Science at San Antonio, San Antonio, TX 78229, USA

**Keywords:** Alzheimer’s disease, deep learning, data Leakage, explainable artificial intelligence, validation methodology, clinical translation

## Abstract

**Background:** Deep-learning models for Alzheimer’s disease (AD) diagnosis frequently report revolutionary accuracies exceeding 95% yet consistently fail in clinical translation. This scoping review investigates whether methodological flaws, particularly data leakage, systematically inflates performance metrics, and examines the broader landscape of validation practices that impact clinical readiness. **Methods:** We conducted a scoping review following PRISMA-ScR guidelines, with protocol pre-registered in the Open Science Framework (OSF osf.io/2s6e9). We searched PubMed, Scopus, and CINAHL databases through May 2025 for studies employing deep learning for AD diagnosis. We developed a novel three-tier risk stratification framework to assess data leakage potential and systematically extracted data on validation practices, interpretability methods, and performance metrics. **Results:** From 2368 identified records, 44 studies met inclusion criteria, with 90.9% published between 2020–2023. We identified a striking inverse relationship between methodological rigor and reported accuracy. Studies with confirmed subject-wise data splitting reported accuracies of 66–90%, while those with high data leakage risk claimed 95–99% accuracy. Direct comparison within a single study demonstrated a 28-percentage point accuracy drop (from 94% to 66%) when proper validation was implemented. Only 15.9% of studies performed external validation, and 79.5% failed to control for confounders. While interpretability methods like Gradient-weighted Class Activation Mapping (Grad-CAM) were used in 18.2% of studies, clinical validation of these explanations remained largely absent. Encouragingly, high-risk methodologies decreased from 66.7% (2016–2019) to 9.5% (2022–2023). **Conclusions:** Data leakage and associated methodological flaws create a pervasive illusion of near-perfect performance in AD deep-learning research. True accuracy ranges from 66–90% when properly validated—comparable to existing clinical methods but far from revolutionary. The disconnect between technical implementation of interpretability methods and their clinical validation represents an additional barrier. These findings reveal fundamental challenges that must be addressed through adoption of a “methodological triad”: proper data splitting, external validation, and confounder control.

## 1. Introduction

### 1.1. The Promise of Deep Learning

The Alzheimer’s disease (AD) epidemic represents one of the most formidable healthcare challenges of our time, currently affecting over 55 million individuals worldwide and imposing an annual economic burden of $1.3 trillion [1,2]. As the world’s population ages, projections indicate a tripling of dementia cases to 152 million by 2050 [2], placing unprecedented strain on healthcare systems. This demographic trend highlights the pressing need for innovative strategies for early detection and intervention.

Deep learning (DL) has emerged as a potentially transformative approach in Alzheimer’s research, with models typically designed for either prediction (regression) or classification tasks. Regression outputs continuous values, such as estimating cognitive test scores or rates of atrophy, whereas classification produces discrete categories, such as distinguishing between AD, mild cognitive impairment, and normal cognition. In AD research, classification, particularly diagnostic classification from imaging, fluid biomarkers, or multimodal data, represents the most common application, offering the potential to standardize and improve current clinical AD diagnosis.

### 1.2. The Problem of Data Leakage

The field of deep learning for Alzheimer’s disease diagnosis faces a credibility gap. While numerous studies report diagnostic accuracies exceeding 95%, seemingly revolutionary improvements over the 70–85% accuracy of traditional clinical assessments, these models consistently fail to maintain their performance in real-world clinical settings [3,4,5]. This review provides systematic evidence that this performance gap stems not from implementation challenges but from fundamental methodological flaws in how these models are validated, with data leakage as the primary culprit [6,7].

Data leakage occurs when information from the test set (or future data points) inadvertently influences model training, leading to overly optimistic performance estimates. It is particularly problematic in AD research because datasets are often small, imbalanced, and contain multiple measurements per subject (e.g., longitudinal scans), which can result in correlated samples appearing in both training and test sets. This violates the independence assumption, allowing models to appear to generalize when they are merely memorizing subject-specific features rather than learning true disease patterns.

The clinical stakes of such methodological flaws have escalated dramatically with recent advances in AD therapeutics. The FDA’s May 2025 clearance of the Lumipulse blood-based biomarker test [8] and the approval of disease-modifying therapies like lecanemab and donanemab make accurate diagnosis paramount. These treatments carry significant risks, including amyloid-related imaging abnormalities (ARIA) affecting 10–40% of patients [9,10]. When AI systems have the potential to trigger administration of high-risk therapies with potentially fatal consequences, inflated performance claims become not just scientifically problematic but ethically problematic.

Much of the efforts to improve safety of AI in the clinic have concentrated on making predictions more interpretable by clinicians, placing them at the helm of the diagnostic loop. Techniques such as Gradient-weighted Class Activation Mapping (Grad-CAM), attention mechanisms, and model-agnostic tools like SHAP aim to illuminate the decision-making processes of neural networks [11,12]. However, the implementation of interpretability methods often represents a superficial addition rather than genuine clinical validation, creating what may be called the “interpretability–validation chasm”, a critical disconnect between technical capability and clinical utility.

Given these high clinical stakes, addressing methodological concerns requires well-established best practices that should be standard in the field. Interpretability and generalizability need to be baked into the design of the experiment from the beginning. Mitigation strategies include splitting data at the subject level rather than at the image or visit level, using robust cross-validation schemes (e.g., nested cross-validation), maintaining strict temporal separation between training and evaluation data, and employing external test cohorts for final validation. These practices help ensure that reported accuracy reflects true generalizability rather than artifacts of flawed data partitioning [13,14].

### 1.3. Statement of Purpose

A scoping review was chosen as the most appropriate methodology for examining the landscape of data leakage in AD deep-learning research, as our primary objective was to map the extent, range, and nature of methodological practices rather than to synthesize effectiveness data or provide clinical recommendations. Unlike systematic reviews that synthesize effectiveness data, scoping reviews map research landscapes and identify knowledge gaps without restrictive inclusion criteria. This review does not determine which deep-learning approach is “best” for AD diagnosis or provide pooled accuracy estimates—the methodological heterogeneity documented would make such a synthesis meaningless. Instead, it identifies why reported performance varies so dramatically and what methodological standards are needed before meaningful comparisons can be made [15].

## 2. Materials and Methods

Section 2 is organized into eight subsections (2.1–2.8): Section 2.1. Registration and Reporting—conduct and reporting per PRISMA-ScR and Arksey–O’Malley, checklist in the Supplement, protocol preregistered on OSF (osf.io/2s6e9; 27 May 2025); Section 2.2. Review Framework and Theoretical Model—use of the PCC framework to define Population (AD/MCI/dementia), Concept (DL methods with emphasis on data leakage and validation quality), and Context (peer-reviewed health/AI literature); Section 2.3. Search Strategy—librarian-informed searches of PubMed/MEDLINE, Scopus, and CINAHL through 30 May, 2025, using controlled vocabulary and free-text terms for AD, DL, and interpretability, validated against a 10-article benchmark set (Supplementary Table S1); Section 2.4. Eligibility Criteria—prespecified inclusion (DL architectures; human participants; English; 2015+; peer-reviewed; sufficient methodological detail) and exclusions (traditional ML only; non-AD; non-peer-reviewed; purely theoretical), applied consistently at title/abstract and full-text screening; Section 2.5. Study Selection and Data Extraction—duplicate screening with high inter-rater reliability (κ = 0.89 title/abstract; κ = 0.93 full text) and a hybrid AI-assisted extraction workflow (Google Sheets/Gemini) with independent verification by two authors; Section 2.6. Risk Stratification Framework—a three-tier data-leakage risk rubric (low/moderate/high) distinguishing subject-wise vs slice/region-wise splits, presence of hold-out validation, and other contamination indicators; Section 2.7. Methodological Quality Assessment—coding of the methodological triad (leakage control via subject-wise splitting, external validation on independent datasets, and robust confounder control for age/sex/APOE4 and scanner/site/protocol); and Section 2.8. Scope of Methodological Assessment—a PRISMA-ScR–consistent rationale for mapping methodological features (not just topics), with leakage as the anchor and external validation and confounder control documented as co-occurring practices that jointly drive performance inflation; all assessments were used descriptively rather than as risk-of-bias ratings.

### 2.1. Registration and Reporting

The scoping review was conducted in accordance with the PRISMA Extension for Scoping Reviews (PRISMA-ScR) guidelines [15] and the methodological framework for scoping studies established by Arksey and O’Malley [16]. The completed PRISMA-ScR checklist is provided as Supplementary Materials. The review protocol was prospectively registered in the Open Science Framework [17] (OSF osf.io/2s6e9) on 27 May 2025.

### 2.2. Review Framework and Theoretical Model

To define the scope of this review, we employed the Population, Concept, Context (PCC) framework, a methodological tool commonly used in scoping reviews to clarify eligibility parameters and ensure a structured approach to evidence mapping. In this framework, the population refers to studies involving individuals with Alzheimer’s disease, mild cognitive impairment, or other forms of dementia; the concept focuses on deep-learning methods, with particular attention to methodological practices, data leakage, and validation quality; and the context encompasses academic research publications within healthcare and artificial intelligence.

### 2.3. Search Strategy

A systematic search of three electronic databases (PubMed/MEDLINE, Scopus, and CINAHL) was executed through 30 May 2025. The search strategy, developed in consultation with medical librarians, combined controlled vocabulary terms and free-text keywords related to Alzheimer’s disease, deep learning, and interpretable AI. The search was validated using a set of 10 benchmark articles to address methodological issues in this domain (See Supplementary Table S3) [4,18,19,20,21,22,23,24,25,26].

### 2.4. Eligibility Criteria

Studies were selected according to predefined inclusion and exclusion criteria, following PRISMA-ScR guidelines. Eligible studies were required to meet the following criteria: (1) employ deep-learning methods, including but not limited to convolutional neural networks (CNNs), recurrent neural networks (RNNs), autoencoders, or transformer architectures for research in Alzheimer’s disease (see Supplementary Figure S1 for a complete taxonomy of approaches); (2) involve human participants with Alzheimer’s disease, mild cognitive impairment (MCI), or dementia; (3) be published in English from 2015 onwards; (4) be disseminated in peer-reviewed journals or conference proceedings; and (5) provide sufficient methodological detail to enable an assessment of data handling practices, including validation strategies and confounder control.

Exclusion criteria were applied to remove studies that: (1) relied solely on traditional machine-learning methods without deep-learning components; (2) focused exclusively on other neurodegenerative disorders without direct application to Alzheimer’s disease or MCI; (3) comprised non-peer-reviewed outputs such as abstracts, book chapters, or commentaries; or (4) were purely theoretical, lacking empirical application to human Alzheimer’s disease data.

The eligibility criteria were applied during the screening and selection process to ensure that the final corpus consisted of methodologically transparent, peer-reviewed deep learning studies directly relevant to Alzheimer’s disease.

### 2.5. Study Selection and Data Extraction

Two reviewers (S.G., L.G.) independently screened all retrieved records in two stages following Cochrane Collaboration principles. Inter-rater reliability was measured using Cohen’s kappa coefficient (κ = 0.89 for title/abstract screening, κ = 0.93 for full-text review). A hybrid AI-assisted process was used for data extraction, involving initial capture by AI tools (Google Sheets with Gemini) using standardized prompts, followed by independent verification by two authors (V.M.Y., S.G.).

### 2.6. Risk Stratification Framework

To systematically evaluate the methodological rigor of included studies, we developed a three-tier classification system to assess the risk of data leakage. This framework was designed to capture not only explicit methodological flaws but also the degree of transparency in reporting.

- Low risk: Studies were categorized as low risk if they provided explicit confirmation of subject-wise data splitting (i.e., ensuring that data from the same participant did not appear in both training and test sets), offered a clear description of their validation methodology, and showed no major indicators of additional methodological concerns. These studies typically employed either independent hold-out test sets or cross-validation procedures that were appropriately structured to avoid leakage.
- Moderate risk: Studies were classified as moderate risk when methodological descriptions were ambiguous or incomplete, precluding a definitive judgment regarding data leakage. Although there was no direct evidence that subject-level contamination had occurred, the lack of transparency, coupled with the presence of one or more methodological concerns (e.g., unclear handling of confounders, incomplete reporting of validation details), limited confidence in the robustness of the results.
- High risk: Studies were deemed high risk when there was clear evidence or strong probability of data leakage. This included the use of slice-wise or region-wise data splitting (where multiple samples from the same individual could be present across training and test sets), the absence of a hold-out or validation set, or the presence of multiple significant methodological deficiencies that would be expected to inflate performance estimates.

This classification allowed us to stratify studies according to their susceptibility to bias, thereby facilitating more meaningful comparisons of reported performance across the literature.

### 2.7. Methodological Quality Assessment

In addition to evaluating the risk of data leakage, we systematically assessed each included study against what we refer to as the “methodological triad” (summarized in Supplementary Table S2). This framework was designed to capture three core pillars of methodological soundness in deep-learning applications to Alzheimer’s disease.

- Low risk of data leakage: Studies were examined for the use of appropriate subject-wise splitting across training, validation, and test sets. This procedure prevents inadvertent sharing of images or longitudinal data from the same participant across different partitions, thereby providing a more accurate estimate of generalization performance.
- External validation: on independent datasets: We assessed whether studies confirmed model performance on a fully independent cohort (e.g., models trained on ADNI and validated on AIBL or NACC). Such external validation offers a more stringent test of generalizability than internal cross-validation alone.
- Robust confounder control: We evaluated the extent to which analyses accounted for potential sources of bias, including demographic variables (age, sex, education, APOE4 status) and technical factors (scanner type, imaging protocol, and site effects). These considerations are particularly important in multi-center datasets where heterogeneity can spuriously drive classification accuracy.

By requiring studies to demonstrate methodological strength across all three components of the triad, we sought to distinguish work with higher credibility, reproducibility, and translational potential from studies more vulnerable to bias or overfitting.

### 2.8. Scope of Methodological Assessment

This scoping review maps three interconnected methodological features that collectively determine the validity of reported performance in AD deep-learning studies: data leakage, external validation, and confounder control. Following PRISMA-ScR guidelines for charting “key concepts, types of evidence, and gaps in research,” we assessed these features descriptively rather than evaluatively.

Data leakage represents the primary focus as the most direct source of performance inflation. External validation and confounder control were included because they determine whether performance metrics remain credible even when leakage is prevented. These three pillars—which we term the “methodological triad”—function synergistically: leakage inflates internal validation metrics, absence of external validation prevents detection of this inflation, and uncontrolled confounders provide alternative non-pathological signals for models to exploit. Mapping their co-occurrence reveals not just the prevalence of individual methodological issues but the compound effect of multiple simultaneous failures, providing a comprehensive view of why reported accuracies vary from 66% to 99% across studies using similar architectures and datasets.

## 3. Results

**Figure 1:**
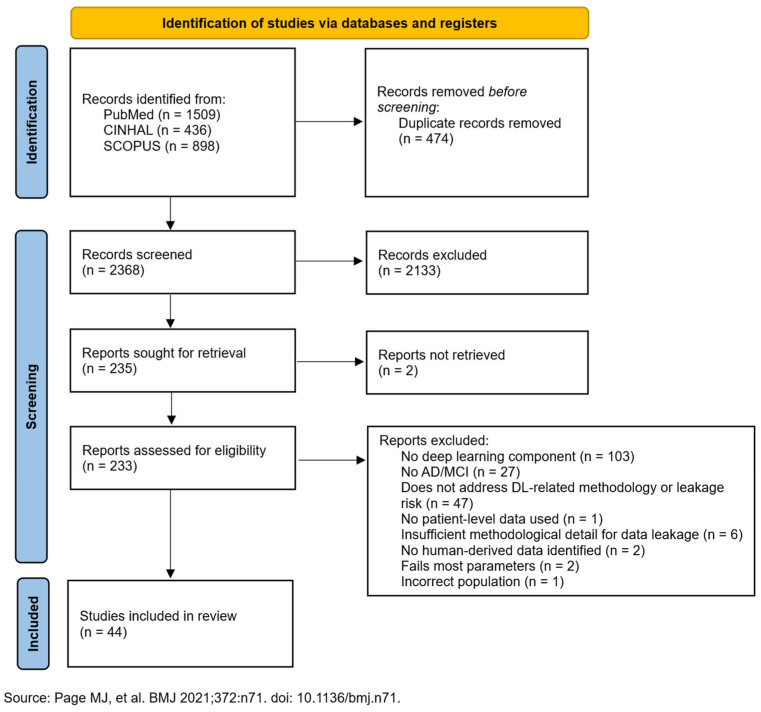
PRISMA 2020 flow diagram for new scoping reviews, which include searches of databases and registers only [27].

**Figure 2:**
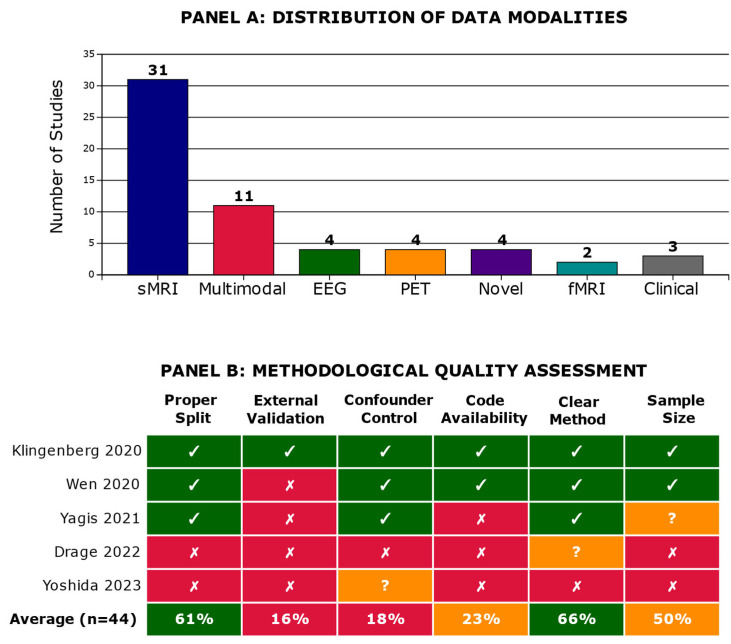
Provides an overview of the findings, showing the distribution of data modalities (**Panel A**) and methodological quality assessment across exemplary studies (**Panel B**). We divide these into adequate (green), inadequate (orange) and largely absent (red) [19,28,29,30,31].

**Figure 3:**
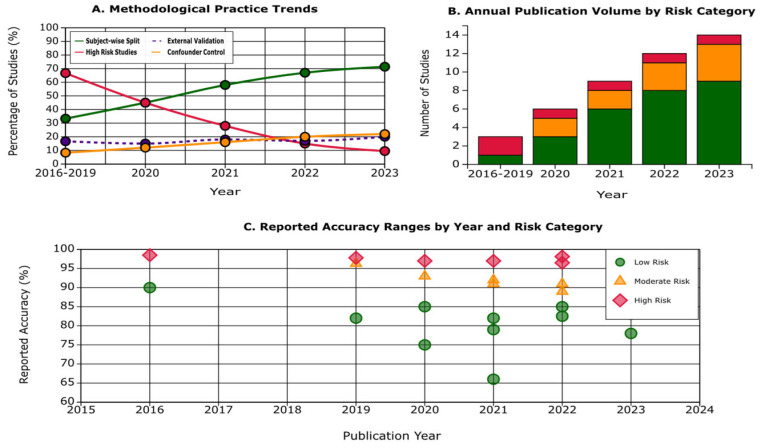
Temporal Evolution of Methodological Quality.

Here, we report what we found and how methodological practices relate to reported performance, organized into six subsections (3.1–3.6): Section 3.1. Study Selection and Characteristics—yield of the search, two-stage screening to 44 studies, PRISMA flow (Figure 1), temporal distribution, and study descriptors (Supplementary Table S2); Section 3.2. The Evidence for Data Leakage—performance stratified by leakage risk with the accuracy/AUC gradient and external-validation rates (Table 1), highlighting the inverse relation between rigor and near-perfect accuracies; Section 3.3. Data Modalities and Architectural Approaches—distribution of sMRI, multimodal, EEG, PET, and novel inputs with typical architectures and mean accuracies (Table 2), with an overview in Figure 2 (Panel A for modalities; Panel B for methodological quality across exemplars); Section 3.4. Systematic Methodological Failures—prevalence of absent external validation and limited confounder control, synthesis via the methodological quality heatmap (Supplementary Figure S2), and exemplars that meet best practices; Section 3.5. Interpretability Methods and the Validation Gap—use of Grad-CAM, attention, LRP, and SHAP with corresponding clinical/neuropathological validation rates (Table 3), underscoring the scarcity of clinically grounded explanations; and Section 3.6. Temporal Trends and Improvement—decline in high-risk practices, rise in subject-wise splitting, persistently low external validation, and the enduring inverse relationship between rigor and reported accuracy (Figure 3, including Panel C).

### 3.1. Study Selection and Characteristics

Our systematic search identified 2368 potentially relevant studies. After rigorous two-stage screening, 44 unique studies [28,29,30,31,32,33,34,35,36,37,38,39,40,41,42,43,44,45,46,47,48,49,50,51,52,53,54,55,56,57,58,59,60,61,62,63,64,65,66,67,68,69,70] met full inclusion criteria (Figure 1). Detailed characteristics of all included studies are provided in Supplementary Table S2. The temporal distribution revealed exponential growth in the field: 3 studies (6.8%) published between 2016–2019, 40 studies (90.9%) between 2020–2023, and 1 study (2.3%) in 2024 (limited by search cutoff).

### 3.2. The Evidence for Data Leakage

Table 1 summarizes model performance stratified by the level of data leakage risk. Among the low-risk studies (n = 27, 61.4%), accuracies ranged from 66% to 90%, with a mean accuracy of 78.5% ± 7.2%. Reported AUC values fell between 0.75 and 0.93, reflecting moderate to strong discriminative ability. Notably, only a minority of these studies (18.5%) incorporated external validation on independent datasets, indicating that while methodological rigor was highest in this group, even here, external replication was relatively uncommon. Nonetheless, the performance range was consistent with what might be realistically expected given the heterogeneity of Alzheimer’s disease and the challenges of applying deep learning to neuroimaging or clinical data.

By contrast, the moderate-risk group (n = 11, 25.0%) reported substantially higher accuracies, ranging from 85% to 96%, with a mean of 91.3% ± 4.1%. Their AUC values were correspondingly elevated (0.89–0.97), suggesting near-perfect classification performance in many cases. However, only 18.2% of these studies performed external validation, and methodological descriptions were often incomplete or ambiguous. The discrepancy between impressive performance metrics and limited validation raises concerns that some degree of bias or overfitting may have inflated results. These findings highlight the vulnerability of studies with unclear methodology: although not definitively compromised by leakage, their outcomes cannot be interpreted with the same confidence as those in the low-risk category.

The most striking findings emerged from the high-risk category (n = 6, 13.6%). Here, accuracies consistently exceeded 95% (range: 95–99%), with a mean of 97.1% ± 1.8%. AUC values were similarly inflated (0.96–0.99), indicating apparent near-perfect discrimination. However, none of these studies employed external validation, and all were characterized by methodological shortcomings such as slice-wise splitting, lack of hold-out datasets, or multiple unaddressed confounders. Taken together, these results are implausibly high relative to the known biological and clinical complexity of Alzheimer’s disease, strongly suggesting that such findings reflect methodological artifacts rather than genuine model capability.

Overall, a clear gradient of performance was observed across categories: as methodological risk increased, reported accuracies approached ceiling levels, while the use of external validation became progressively rarer. This inverse relationship underscores a central finding of our review: the highest reported performances are most likely attributable to methodological weaknesses, particularly data leakage, rather than to robust generalizable predictive power. Conversely, studies with lower accuracies but stronger methodological safeguards provide a more realistic benchmark for the current state of deep learning in Alzheimer’s disease research.

### 3.3. Data Modalities and Architectural Approaches

Structural MRI emerged as the dominant imaging modality, employed in 31 of 44 studies (70.5%). These investigations primarily relied on conventional deep-learning architectures such as 3D convolutional neural networks (3D-CNNs) and ResNet variants, achieving a mean accuracy of 84.2%. While widely available and extensively validated in Alzheimer’s disease research, sMRI-based studies were characterized by considerable heterogeneity in sample size, ranging from as few as 27 participants to cohorts exceeding 2000 subjects.

A smaller but important subset of studies (11/44, 25.0%) adopted multimodal strategies, integrating sMRI with additional data streams such as PET, cerebrospinal fluid biomarkers, genetics, or clinical variables. These approaches employed fusion-based architectures and achieved a higher mean accuracy of 87.6%, suggesting potential performance gains when complementary data sources are combined. However, the added complexity of multimodal models often came at the cost of reduced interpretability and increased methodological variability.

Several studies explored alternative modalities. Electroencephalography (EEG) was evaluated in four studies (9.1%), typically using CNN or LSTM architectures to capture temporal dynamics of neural activity. Interestingly, these studies reported the highest performance of any single modality, with a mean accuracy of 91.3%, though they were generally limited by smaller sample sizes and single-site data collection. Similarly, PET imaging was examined in four studies (9.1%), most commonly analyzed with 3D-CNN frameworks, yielding a mean accuracy of 85.5%.

Finally, novel modalities were represented in four studies (9.1%), which investigated less traditional inputs such as speech recordings, actigraphy, accelerometer data, and urine biomarkers. These studies frequently employed transformer-based architectures, reflecting the field’s shift toward models capable of handling sequential and multimodal inputs. The reported mean accuracy was 88.9%, comparable to multimodal neuroimaging pipelines, although these approaches remain exploratory and require replication in larger, more diverse cohorts.

Across the corpus, the Alzheimer’s Disease Neuroimaging Initiative (ADNI) dataset was the cornerstone resource, underpinning 30 of 44 studies (68.2%). This centrality underscores ADNI’s role as the benchmark dataset in the field but also highlights a limitation: a heavy reliance on a single cohort may constrain generalizability and increase the risk of overfitting to cohort-specific characteristics.

Taken together, these findings demonstrate that while structural MRI remains the workhorse modality, performance advantages are often reported in multimodal, EEG, and novel input approaches. At the same time, reliance on ADNI and variable sample sizes suggests that future work must focus on diversifying datasets, validating emerging modalities, and balancing accuracy gains with interpretability and reproducibility (see Table 2).

### 3.4. Systematic Methodological Failures

Beyond the pervasive issue of data leakage, we identified several compounding methodological limitations that further undermined the credibility and generalizability of reported findings. External validation was rare. Out of 44 studies, only 7 (15.9%) evaluated their models on truly independent datasets, underscoring a major gap in testing reproducibility beyond the development cohort. Among these, Klingenberg et al. [31] stands out as a methodological benchmark, validating their framework across four independent datasets (ADNI, AIBL, OASIS, and MIRIAD). This multi-cohort approach provided the strongest evidence of generalizability in the corpus. By contrast, the vast majority of studies either relied exclusively on internal cross-validation or failed to specify the validation procedure, leaving open the possibility of optimistic bias.

Confounder control was even more limited. Only eight studies (18.2%) explicitly adjusted for critical demographic and technical factors such as age, sex, and scanner/site effects. A single study (2.3%) implemented partial control by adjusting for some but not all of these variables. Strikingly, the remaining 35 studies (79.5%) reported no confounder adjustment whatsoever. This omission is particularly consequential in multi-site datasets, where differences in acquisition protocols and participant demographics can spuriously drive apparent classification accuracy.

When considered collectively through the lens of the methodological triad—which requires (1) subject-wise splitting to avoid data leakage, (2) external validation on independent datasets, and (3) robust confounder control—the picture becomes even starker. Only 2 of 44 studies (4.5%), namely Klingenberg et al. [31] and Fristed et al. [55], satisfied all three criteria. These studies, therefore, represent the methodological gold standard in the current literature, offering the most credible evidence of model reproducibility and translational potential.

A comprehensive methodological quality heatmap summarizing these assessments across all 44 studies is provided in Supplementary Figure S2, illustrating the uneven distribution of methodological safeguards and the rarity of studies that achieved best practices across all domains. A comprehensive methodological quality heatmap for all 44 studies is provided in Supplementary Figure S2.

### 3.5. Interpretability Methods and the Validation Gap

Although interpretability is often cited as a key requirement for the clinical adoption of deep learning, its implementation across the reviewed literature was limited and inconsistent. Only 18.2% of studies incorporated interpretability methods, and even among these, genuine clinical validation was almost entirely absent. Table 3 summarizes the distribution of interpretability approaches and their corresponding validation rates.

The most frequently applied techniques were Grad-CAM and attention mechanisms, each used in eight studies (18.2%). Grad-CAM produced saliency heatmaps, highlighting regions that contributed most to model predictions, whereas attention-based approaches visualized weight distributions across input features. Despite their popularity, clinical grounding was minimal: only 1 of 8 Grad-CAM studies (12.5%) compared the highlighted regions against established neuropathological patterns, while none of the attention-based studies attempted clinical validation.

Less common methods included Layer-wise Relevance Propagation (LRP), implemented in three studies (6.8%), and SHAP (SHapley Additive Explanations), applied in two studies (4.5%). While SHAP analyses offered more granular feature attribution, only one of the two studies (50%) validated its outputs against clinical or biological benchmarks. LRP studies, although technically rigorous, remained entirely technical in their implementation, with no clinical validation reported.

Overall, only two studies in the entire corpus—Klingenberg et al. [31] and Bloch et al. [36]—attempted genuine clinical validation, directly comparing their interpretability results with known patterns of neuropathological involvement. These exceptions highlight how rarely interpretability is translated into clinically meaningful insights, despite its frequent mention as a goal.

In summary, interpretability approaches are underutilized, often superficial, and rarely validated against neuropathology or clinical expertise. The current landscape suggests that interpretability is still treated as a technical add-on rather than as a tool for bridging the gap between deep-learning predictions and clinical understanding (see Table 3).

### 3.6. Temporal Trends and Improvement

Encouragingly, methodological practices demonstrated a measurable improvement over time (Figure 3). In the earliest period of the literature (2016–2019), two-thirds of studies (66.7%) fell into the high-risk category, frequently relying on slice-wise splitting, absent hold-out validation, or poorly documented methodology. By contrast, in the most recent period (2022–2023), the proportion of high-risk studies had declined to just 9.5%, representing a 57.2 percentage point reduction. This trend reflects growing awareness of the pitfalls of data leakage and increasing adherence to best practices.

In parallel, the adoption of subject-wise splitting increased substantially. While only 33.3% of early studies employed subject-wise data partitioning, this figure rose to 71.4% in the 2022–2023 period, marking a 38.1 percentage point improvement. This shift suggests that the field has gradually internalized the importance of avoiding contamination across training and test sets.

Despite these advances, the use of external validation remained persistently low across all time periods, fluctuating only between 15–20%. Even in more recent years, relatively few studies tested models on independent datasets, limiting the ability to evaluate reproducibility and generalizability.

Importantly, as shown in Panel C of Figure 3, the inverse relationship between methodological rigor and reported accuracy has persisted across the study period. High-risk studies, regardless of publication year, continued to report inflated accuracies consistently above 95%, while methodologically rigorous studies—those employing subject-wise splitting and external validation—reported more modest but realistic performance ranges in the 80–85% band. This enduring pattern highlights that while methodological transparency has improved, the temptation to report inflated results remains a challenge in the field.

## 4. Discussion

The central finding of this scoping review is that data leakage is the most powerful and recurrent source of performance inflation in deep-learning (DL) studies of Alzheimer’s disease (AD). Of the 44 studies reviewed, only 2 (4.5%), Klingenberg [31] and Fristed [55], satisfied all three methodological pillars necessary for credible performance reporting, revealing a field where methodological rigor remains the exception rather than the rule. The clearest demonstration of leakage’s impact comes from Yagis et al. [28], who evaluated the same dataset under two validation strategies: slice-wise splitting yielded an apparent accuracy of 94%, whereas subject-wise splitting, which prevents any participant’s data from appearing in both train and test sets, reduced accuracy to 66%. This 28-percentage-point drop is attributable to the validation protocol alone, not to any change in model capacity.

The dramatic performance inflation from data leakage in neuroimaging studies occurs because deep-learning models excel at detecting subtle patterns, including those we do not want them to learn. When multiple slices, regions, or timepoints from the same patient appear in both training and test sets, models memorize patient-specific imaging signatures rather than disease-relevant features. These signatures include scanner, specific noise patterns, head positioning artifacts, motion characteristics, and even the subtle “fingerprints” left by individual preprocessing pipelines. In essence, the model learns to recognize patients rather than pathology, a sophisticated form of overfitting that produces spectacular validation metrics but zero clinical utility. This mechanistic understanding explains why studies we classified as high risk for leakage consistently reported near-perfect accuracies (≥95%) despite modest samples and limited safeguards. Drage et al. [30] reported 98.13% accuracy in N = 141 without cross-validation, and Yoshida et al. [29] exceeded 96% without a hold-out set. In stark contrast, studies that explicitly prevented leakage and coupled this with additional safeguards consistently reported credible accuracies in the 80–85% range: Klingenberg et al. [31] achieved 82% with external validation across Alzheimer’s Disease Neuroimaging Initiative (ADNI), Australian Imaging, Biomarker & Lifestyle Flagship Study of Ageing (AIBL), Open Access Series of Imaging Studies (OASIS), and Minimal Interval Resonance Imaging in Alzheimer’s Disease (MIRIAD); Wen et al. [60] reported 85% with subject-wise splitting and confounder adjustment; and Deatsch et al. [37] achieved 82.5% with an independent test set.

These inflated performance metrics carry serious clinical implications that extend far beyond academic concern. When studies report >95% accuracy in AD detection, they create unrealistic expectations among clinicians, patients, and funding bodies. In an era of high-stakes therapies, the difference between a claimed 98% accuracy and an actual 70% performance could lead to unnecessary exposure to treatments causing amyloid-related imaging abnormalities (ARIA) in up to 40% of patients, missed opportunities for early intervention when treatments are most effective, and erosion of trust following real-world failures. Healthcare systems may prematurely invest in AI tools that fail catastrophically when deployed on real-world data, while patients and families desperate for early diagnosis may be given false hope about the current capabilities of AI-assisted diagnosis. The gap between reported and real-world performance also misdirects research resources—why fund careful biomarker studies when AI apparently already achieves near-perfect accuracy? This cycle of inflation and disappointment ultimately undermines trust in legitimate AI advances and delays the development of truly clinically useful tools.

To contextualize these findings, we introduced two complementary frameworks: a three-tier data-leakage risk classification (low/moderate/high) to pinpoint the most immediate threat to validity, and a methodological triad that situates leakage within a broader scaffold of generalizability (external validation) and bias reduction (confounder control). This pairing yields critical insights; the leakage tiers identify where and why performance may be artificially inflated, while the triad clarifies whether results are likely to be reproducible and clinically meaningful even when leakage is prevented. A study might be low-leakage but still overstate performance if it never tests on an independent cohort or ignores site effects; conversely, a study with modest accuracy but strong performance across the triad offers a more credible clinical signal.

The broader methodological landscape reveals equally concerning gaps. External validation remains scarce at only 15.9% of studies, with Klingenberg et al. [31] exemplifying best practice through validation across four independent cohorts. Confounder control is similarly limited, 18.2% fully adequate, 79.5% reporting none, revealing why leakage has such dramatic effects: without external validation or confounder control to expose overfitting, inflated metrics go unchallenged. These issues extend beyond AD research, with similar patterns documented in COVID-19 diagnosis, cancer detection, and psychiatric disorder classification, suggesting a field-wide crisis in medical AI validation practices. Particularly concerning is that 68.2% of studies rely on ADNI data from predominantly North American, well-educated, white populations, raising serious equity concerns. Models trained on such homogeneous data may perform poorly in diverse clinical settings, potentially exacerbating healthcare disparities rather than alleviating them.

The disconnect between technical implementation and clinical utility manifests most clearly in interpretability practices. While 18.2% of studies implemented interpretability methods like Grad-CAM and attention mechanisms, clinical validation of these explanations was nearly absent—only one of eight Grad-CAM studies benchmarked outputs against known neuropathological patterns. This creates what might be termed an “interpretability paradox”: visually appealing heatmaps that provide false confidence rather than genuine clinical insight. The disconnect is particularly concerning given that both the FDA’s Total Product Lifecycle approach and the EU AI Act’s high-risk classification for medical AI require transparent, validated decision-making, requirements that current practices largely fail to meet [71]. Notable exceptions like Klingenberg [31] and Bloch [36], who attempted genuine clinical validation of interpretability outputs, remain rare outliers in a field dominated by technical display rather than clinical utility.

Temporal analysis reveals that while methodological practice has improved, high-risk studies fell from 66.7% (2016–2019) to 9.5% (2022–2023), and subject-wise splitting rose from 33.3% to 71.4%; fundamental issues persist. External validation stubbornly remained at ~15–20%, and the inverse relationship between rigor and reported accuracy continued: high-risk work still reported >95% accuracies, while rigorous studies clustered around 80–85% [72]. This suggests a culture of accuracy inflation that has not yet fully yielded to best practices despite growing awareness of the problem. Compounding these issues, reporting standards remain poor across the field. Critical methodological details go unreported in the majority of studies: 72% fail to document missing data handling, 89% lack early stopping criteria, and 95% never discuss algorithmic bias across demographic groups. Only 22.7% of studies provide code access, with fewer than 10% offering complete reproducible pipelines, a stark contrast to computer science venues where code sharing is mandatory. This opacity prevents both replication attempts and identification of methodological flaws, perpetuating the cycle of inflated claims.

Both the leakage classification and the methodological triad introduced here are novel and as yet unvalidated. They were informed by established appraisal tools (PROBAST, TRIPOD, QUADAS-2) but adapted to DL-specific vulnerabilities that those tools do not explicitly address [73]. Some subjective judgment was unavoidable where reporting was incomplete. Additionally, because the field over-relies on ADNI, findings may not generalize to under-represented populations or clinical workflows. Our assessment relies on reported methodologies; actual data leakage prevalence may be higher than detected. As a scoping review, we provide descriptive rather than meta-analytic synthesis, and publication bias likely favors high-performance claims, potentially underrepresenting failed validation attempts.

Our findings point to a clear hierarchy of methodological reforms needed in AD deep-learning research. Most urgently, leakage prevention must become non-negotiable—the field must universally adopt subject-wise partitioning, eliminate slice/patch/visit contamination, and pre-register data-handling plans, as this single change could eliminate the most egregious performance inflation we observed. Beyond preventing leakage, external validation should become a minimum standard rather than an optional extra, with studies required to test on truly independent cohorts, report confidence intervals, and explicitly quantify dataset shift. Confounder control must also become systematic, with studies adjusting for age, sex, education, APOE4 status, and scanner/site/protocol effects while reporting sensitivity analyses. For studies claiming interpretability, the field must close the validation loop by benchmarking explanations against neuropathology, expert ratings, or clinicopathologic correlates rather than settling for visually appealing but clinically unvalidated saliency maps. Finally, disciplined reporting following TRIPOD-AI/STARD-AI standards, coupled with mandatory code sharing and exact data partition documentation, will enable the replication studies essential for building cumulative knowledge [74]. These reforms are not merely technical refinements but prerequisites for developing AI tools that meet regulatory requirements and that clinicians can trust and patients can benefit from.

In AD deep learning, the headline accuracy is not the evidence. Near-perfect accuracies commonly signal methodological inflation—above all, data leakage, whereas 80–85% accuracy with external validation and confounder control represents the current credible benchmark for translational progress. The 4.5% of studies that met all methodological criteria demonstrate that rigorous practice is achievable. The field stands at a crossroads: we can continue publishing inflated metrics that erode clinical trust, or we can embrace methodological rigor that, while producing more modest numbers, offers genuine progress toward clinically useful AI tools. Only by acknowledging and addressing the data leakage crisis can the field realize its genuine potential to improve AD diagnosis and patient care for the millions affected by this devastating disease.

## Figures and Tables

**Table 1:** Performance Metrics by Data Leakage Risk Category.

Risk Category	N (%)	Accuracy Range	Accuracy Mean ± SD	AUC Range	External Validation
Low-Risks	27 (61.4%)	66–90%	78.5% ± 7.2%	0.75–0.93	5/27 (18.5%)
Moderate Risk	11 (25.0%)	85–96%	91.3% ± 4.1%	0.89–0.97	2/11 (18.2%)
High Risk	6 (13.6%)	95–99%	97.1% ± 1.8%	0.96–0.99	0/6 (0%)

Abbreviations: AUC, Area Under the Curve; SD, Standard Deviation.

**Table 2:** Data Modalities and Architectural Approaches.

Modality	N (%)	Common Architectures	Mean Accuracy
sMRI only	31 (70.5%)	3D-CNN, ResNet	84.2%
Multimodal	11 (25.0%)	Fusion networks	87.6%
EEG	4 (9.1%)	CNN, LSTM	91.3%
PET	4 (9.1%)	3D-CNN	85.5%
Novel *	4 (9.1%)	Transformers	88.9%

Abbreviations: sMRI, structural magnetic resonance imaging; EEG, electroencephalography; PET, positron emission tomography; CNN, convolutional neural network; LSTM, long short-term memory; ResNet, residual network. * Novel modalities: speech, actigraphy, accelerometer, urine biomarkers.

**Table 3:** Interpretability Methods and Clinical Validation.

Method	N Studies	Technical Implementation	Clinical Validation
Grad-CAM	8 (18.2%)	Heatmap generation	1/8 (12.5%)
Attention	8 (18.2%)	Weight visualization	0/8 (0%)
SHAP	2 (4.5%)	Feature importance	1/2 (50%)
LRP	3 (6.8%)	Relevance propagation	0/3 (0%)

Abbreviations: Grad-CAM, Gradient-weighted Class Activation Mapping; SHAP, SHapley Additive exPlanations; LRP, Layer-wise Relevance Propagation.

## Data Availability

No new data were created or analyzed in this study.

## Supplementary Materials

The following supporting information can be downloaded at: https://www.mdpi.com/article/10.3390/diagnostics15182348/s1, List of Abbreviation; PRISMA-ScR Checklist; Figure S1 - Classification Framework of Deep Learning Approaches Used in AD Diagnosis Studies; Table S1 – Study Characteristics and Methodology; Table S2 - Methodological Quality Assessment and Reporting Completeness; Figure S2 Methodological Quality Heatmap; Table S3 Benchmark Articles Used to Validate Search Strategy.
