# Supplementary material for: Data Leakage in Deep Learning for Alzheimer’s Disease Diagnosis: A Scoping Review of Methodological Rigor and Performance Inflation
Source: Diagnostics (Basel). 2025 Sep 16;15(18):2348. doi: 10.3390/diagnostics15182348 (PMC12468286; doi:10.3390/diagnostics15182348)
Supplement: Supplementary file 1 [file diagnostics-15-02348-s001.zip › diagnostics-3761005-supplementary.pdf]

List of Abbreviations

Abbreviation	Full Term
AD	Alzheimer’s Disease
ADNI	Alzheimer's Disease Neuroimaging Initiative
AI	Artificial Intelligence
ARIA	Amyloid-Related Imaging Abnormalities
AUC	Area Under the Curve
CC BY	Creative Commons Attribution
CINAHL	Cumulative Index to Nursing and Allied Health Literature
CNNs	Convolutional Neural Networks
DL	Deep Learning
EEG	Electroencephalogram
EU	European Union
FDA	Food and Drug Administration
Grad-CAM	Gradient-weighted Class Activation Mapping
LRP	Layer-wise Relevance Propagation
LSTM	Long Short-Term Memory
MCI	Mild Cognitive Impairment
MRI	Magnetic Resonance Imaging
OSF	Open Science Framework
PCC	Population, Concept, Context
PET	Positron Emission Tomography
PRISMA-ScR	Preferred Reporting Items for Systematic Reviews and Meta-Analyses extension for Scoping Reviews
RNNs	Recurrent Neural Networks
SD	Standard Deviation
SHAP	SHapley Additive exPlanations
sMRI	structural Magnetic Resonance Imaging
STARD-AI	STandards for the Reporting of Diagnostic accuracy studies-Artificial Intelligence
TRIPOD-AI	Transparent Reporting of a multivariable prediction model for Individual Prognosis Or Diagnosis-Artificial Intelligence

Preferred Reporting Items for Systematic reviews and Meta-Analyses extension for Scoping Reviews (PRISMA-ScR) Checklist

SECTION	ITEM	PRISMA-ScR CHECKLIST ITEM	REPORTED ON PAGE #
**TITLE**			
Title	1	Identify the report as a scoping review.	1
**ABSTRACT**			
Structured summary	2	Provide a structured summary that includes (as applicable): background, objectives, eligibility criteria, sources of evidence, charting methods, results, and conclusions that relate to the review questions and objectives.	1
**INTRODUCTION**			
Rationale	3	Describe the rationale for the review in the context of what is already known. Explain why the review questions/objectives lend themselves to a scoping review approach.	1-3
Objectives	4	Provide an explicit statement of the questions and objectives being addressed with reference to their key elements (e.g., population or participants, concepts, and context) or other relevant key elements used to conceptualize the review questions and/or objectives.	3
**METHODS**			
Protocol and registration	5	Indicate whether a review protocol exists; state if and where it can be accessed (e.g., a Web address); and if available, provide registration information, including the registration number.	4
Eligibility criteria	6	Specify characteristics of the sources of evidence used as eligibility criteria (e.g., years considered, language, and publication status), and provide a rationale.	4-5
Information sources*	7	Describe all information sources in the search (e.g., databases with dates of coverage and contact with authors to identify additional sources), as well as the date the most recent search was executed.	5
Search	8	Present the full electronic search strategy for at least 1 database, including any limits used, such that it could be repeated.	5
Selection of sources of evidence†	9	State the process for selecting sources of evidence (i.e., screening and eligibility) included in the scoping review.	5-6
Data charting process‡	10	Describe the methods of charting data from the included sources of evidence (e.g., calibrated forms or forms that have been tested by the team before their use, and whether data charting was done independently or in duplicate) and any processes for obtaining and confirming data from investigators.	5-6
Data items	11	List and define all variables for which data were sought and any assumptions and simplifications made.	5-6
Critical appraisal of individual sources of evidence§	12	If done, provide a rationale for conducting a critical appraisal of included sources of evidence; describe the methods used and how this information was used in any data synthesis (if appropriate).	5-6
Synthesis of results	13	Describe the methods of handling and summarizing the data that were charted.	5-6

SECTION	ITEM	PRISMA-ScR CHECKLIST ITEM	REPORTED ON PAGE #
**RESULTS**			
Selection of sources of evidence	14	Give numbers of sources of evidence screened, assessed for eligibility, and included in the review, with reasons for exclusions at each stage, ideally using a flow diagram.	6-7
Characteristics of sources of evidence	15	For each source of evidence, present characteristics for which data were charted and provide the citations.	6-15
Critical appraisal within sources of evidence	16	If done, present data on critical appraisal of included sources of evidence (see item 12).	15-20
Results of individual sources of evidence	17	For each included source of evidence, present the relevant data that were charted that relate to the review questions and objectives.	15-20
Synthesis of results	18	Summarize and/or present the charting results as they relate to the review questions and objectives.	15-20
**DISCUSSION**			
Summary of evidence	19	Summarize the main results (including an overview of concepts, themes, and types of evidence available), link to the review questions and objectives, and consider the relevance to key groups.	21-24
Limitations	20	Discuss the limitations of the scoping review process.	25
Conclusions	21	Provide a general interpretation of the results with respect to the review questions and objectives, as well as potential implications and/or next steps.	25
**FUNDING**			
Funding	22	Describe sources of funding for the included sources of evidence, as well as sources of funding for the scoping review. Describe the role of the funders of the scoping review.	26

JBI = Joanna Briggs Institute; PRISMA-ScR = Preferred Reporting Items for Systematic reviews and Meta-Analyses extension for Scoping Reviews.
* Where *sources of evidence* (see second footnote) are compiled from, such as bibliographic databases, social media platforms, and Web sites.
† A more inclusive/heterogeneous term used to account for the different types of evidence or data sources (e.g., quantitative and/or qualitative research, expert opinion, and policy documents) that may be eligible in a scoping review as opposed to only studies. This is not to be confused with *information sources* (see first footnote).
‡ The frameworks by Arksey and O'Malley (6) and Levac and colleagues (7) and the JBI guidance (4, 5) refer to the process of data extraction in a scoping review as data charting.
§ The process of systematically examining research evidence to assess its validity, results, and relevance before using it to inform a decision. This term is used for items 12 and 19 instead of "risk of bias" (which is more applicable to systematic reviews of interventions) to include and acknowledge the various sources of evidence that may be used in a scoping review (e.g., quantitative and/or qualitative research, expert opinion, and policy document).

From: Tricco AC, Lillie E, Zarin W, O'Brien KK, Colquhoun H, Levac D, et al. PRISMA Extension for Scoping Reviews (PRISMAScR): Checklist and Explanation. Ann Intern Med. 2018;169:467–473. doi: 10.7326/M18-0850.

Figure S1 Classification Framework of Deep Learning Approaches Used in AD Diagnosis Studies

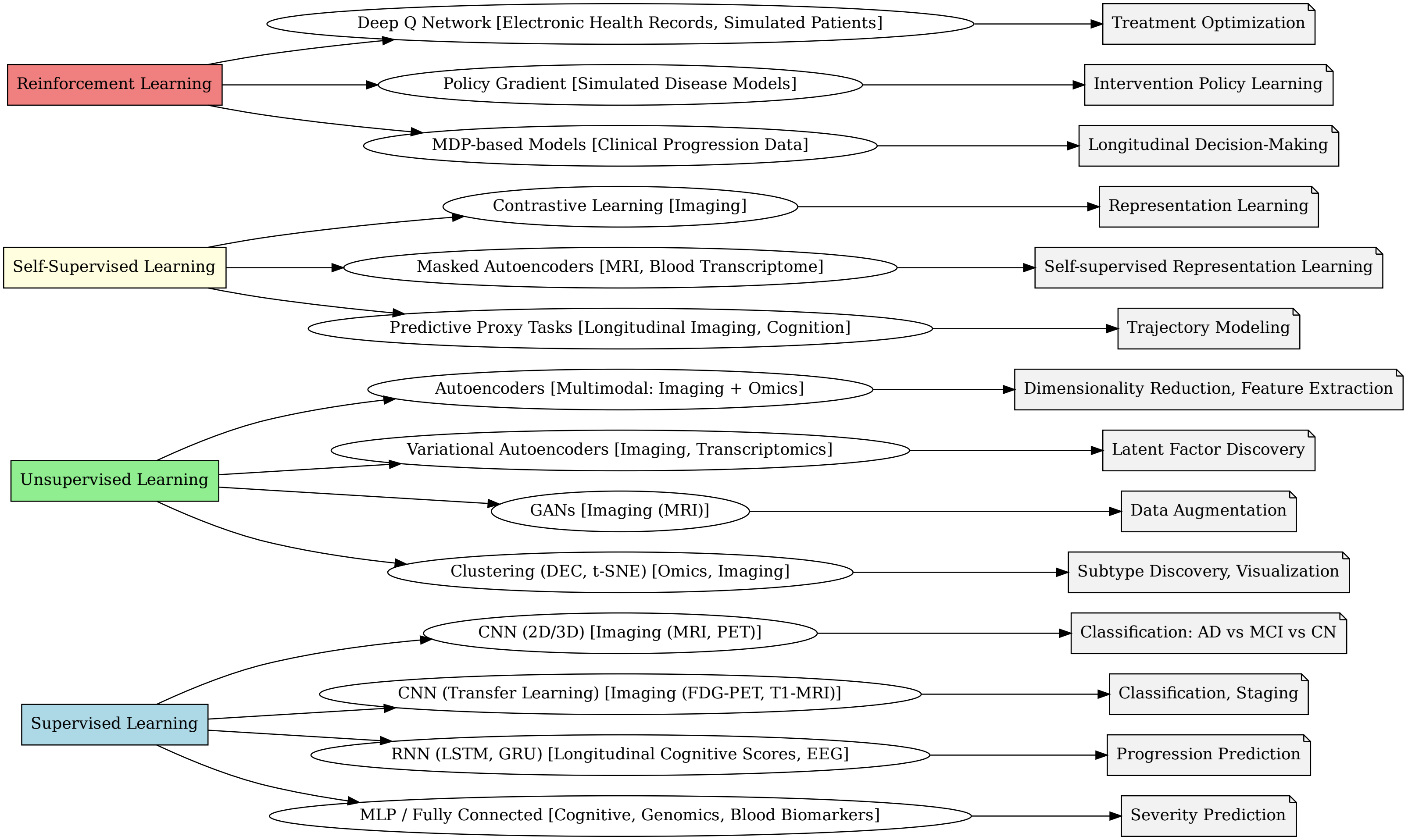

Table S1. Summary of Study Characteristics and Methodology

Author, Year	Data Modality	Task Type	n	Dataset	Validation Strategy	Data Leakage Risk	External Validation	Confounder Control Reported
Santos Bringas et al., 2023	Accelerometer	Classification	35	In-house	Not Reported	LOW	NO	NR
Ruwanpathirana et al., 2022	Tau-PET	Regression	134	In-house	10-fold CV	LOW	NO	YES (age, sex)
Yagis et al., 2021	sMRI	Classification	200+	OASIS, ADNI, PPMI	Nested 5-fold CV	LOW	NO	YES (age, sex)
Rutkowski et al., 2023	EEG	Classification	30	In-house	LOOCV	LOW	NO	NR
Ortiz et al., 2016	sMRI	Classification	818	ADNI	k-fold CV	LOW	NO	NR
Bloch et al., 2024	sMRI	Classification	380	ADNI	Hold-out	LOW TO MODERATE	YES	NR
Deatsch et al., 2022	sMRI, PET	Classification	772+	ADNI	Independent Test Set	LOW TO MODERATE	YES	YES (age, gender, scanner, site)
Mahendran et al., 2022	sMRI	Classification	402	ADNI	10-fold CV	MODERATE	NO	NR
Yoshida et al., 2023	Urine Biomarkers	Classification	161	In-house	No CV/Hold-out	HIGH	NO	PARTIAL (age)
Drage et al., 2022	EEG	Classification	141	In-house	Hold-out (70/30)	HIGH	NO	NR
Srivishagan et al., 2022	sMRI, Neuropsych	Classification	736	ADNI	5-fold CV	LOW	NO	YES (age, sex)
Tsang et al., 2019	sMRI	Classification	400	ADNI	5-fold CV (unclear)	MODERATE	NO	NR
Bit et al., 2021	sMRI	Classification	750	ADNI	10-fold CV	LOW	YES	NR
Rutkowski et al., 2022	sMRI, DTI	Classification	846	ADNI	5-fold CV	LOW	NO	NR
Ozkaraca et al., 2021	sMRI, Clinical	Classification	843	ADNI	10-fold CV	LOW	NO	NR
Cardenas-Peña et al., 2021	sMRI, Clinical	Classification	737	ADNI	10-fold CV	MODERATE	NO	NO
Amezquita-Sanchez et al., 2021	EEG	Classification	44	Private	5-fold CV	MODERATE	NO	NR
Zaman et al., 2022	sMRI, Clinical	Classification	867	ADNI	10-fold CV (unclear)	MODERATE	NO	NR
Neira-Rodado et al., 2023	sMRI	Classification	400	Private	5-fold CV (unclear)	MODERATE	NO	NR
Alarjani et al., 2023	sMRI, Genetic	Classification	800	ADNI	5-fold CV (unclear)	HIGH	NO	NO
Park et al., 2019	sMRI	Classification	819	Private	5-fold CV	LOW	YES (ADNI)	NO
Kar et al., 2021	sMRI	Classification	640	ADNI	5-fold CV (unclear)	HIGH	NO	NO

Gong et al., 2021	sMRI, Clinical	Classification	1,054	ADNI	10-fold CV (unclear)	HIGH	NO	NO
Gyawali et al., 2022	sMRI	Classification	798	ADNI	5-fold CV (unclear)	HIGH	NO	NO
Guelib et al., 2022	sMRI	Classification	400	ADNI	5-fold CV	LOW	NO	NR
Sethuraman et al., 2023	sMRI, Clinical	Classification	1,028	ADNI	5-fold CV (unclear)	MODERATE	NO	NO
Fristed et al., 2021	Speech	Classification	133	Private	Train/Test Split	LOW	YES (BioFINDER, ADNI)	YES (age, sex, scanner)
Chang et al., 2023	fMRI	Classification	288	ADNI	10-fold CV (unclear)	MODERATE	NO	NO
Zhang et al., 2021	sMRI	Classification	373	ADNI	5-fold CV (unclear)	MODERATE	NO	NR
Basheera & Ram, 2021	sMRI	Classification	820	ADNI	10-fold CV	LOW	YES (OASIS)	NR
Akhtar et al., 2022	Neuropsych	Regression/Class.	246	ADNI	5-fold CV	LOW	NO	YES (age, sex, scanner)
Wen et al., 2020	sMRI	Classification	>2,000 subjects	ADNI	5-fold CV	LOW	NO	YES (site, age, sex)
Choi et al., 2022	Neuropsych, Clinical	Prediction	921	ADNI	Hold-out	LOW	NO	NR
Gallucci et al., 2023	Actigraphy	Classification	88	In-house	5-fold CV	LOW	NO	NR
Dong et al., 2020	sMRI	Classification	858	ADNI	5-fold CV	LOW	NO	NR
Jo et al., 2020	PET, sMRI	Classification	300	ADNI	5-fold CV	LOW	NO	NR
Klingenberg et al., 2020	sMRI	Classification	361	ADNI	10-fold CV	LOW	YES (AIBL, OASIS, MIRIAD)	YES (age, sex, MMSE, scanner)
Bi et al., 2020	sMRI, PET	Classification	200	ADNI	10-fold CV	LOW	NO	NR
Luo et al., 2022	sMRI, fMRI	Classification	420	ADNI	5-fold CV	LOW	NO	NR
Han et al., 2021	sMRI	Classification	377	ADNI	5-fold CV	LOW	NO	NR
Irie et al., 2020	sMRI	Classification	69	In-house	LOOCV	LOW	NO	NR
Ntracha et al., 2022	sMRI	Classification	408	ADNI	10-fold CV	LOW	NO	NR
Song et al., 2021	sMRI	Classification	326	ADNI	5-fold CV	LOW	NO	NR
Xing et al., 2021	PET	Classification	381	ADNI	5-fold CV	LOW	NO	NR

Abbreviations: CV: Cross-Validation; LOOCV: Leave-One-Out Cross-Validation; sMRI: structural Magnetic Resonance Imaging; DTI: Diffusion Tensor Imaging; fMRI: functional Magnetic Resonance Imaging; PET: Positron Emission Tomography; EEG: Electroencephalography; Neuropsych: Neuropsychological; NR: Not Reported.

Table S2. Methodological Quality Assessment and Reporting Completeness

Author, Year	Preprocessing Steps Described	Model Architecture Described	Validation Strategy Described	Data Splitting Level	Data Leakage Addressed or Avoided	Confounder Control Reported	External Validation Performed	Reporting Completeness	Overall Data Leakage Risk	Additional Methodological Concerns or Flags
Santos Bringas et al., 2023	**YES**	YES	YES	subject-wise	YES	NR	NO	MOD	LOW	Small sample size with class imbalance, no demographic adjustment, no external validation, no open access to code or data,
Ruwanpathirana et al., 2022	**YES**	NR	YES	subject-wise	YES	YES (age, sex)	NO	MOD	LOW	Model details limited, with no codes or weights provided. No external validation and validation/test sets are small; Class imbalance and regularization not clearly addressed. Confounder control limited to age and sex.
Yagis et al., 2021	**YES**	YES	YES	subject-wise & splice-wise	YES	YES (age, sex)	NO	HIGH	LOW	Overfitting in subject-level CV reported, no harmonization across MRI scanner vendors/sites (although variation acknowledged), no external test set, small datasets limit statistical power.
Rutkowski et al., 2023	**YES**	YES	YES	subject-wise	YES	NR	NO	MOD	LOW	Small sample size, subjective MoCA cutoff used for labeling MCI (less than/equal to 25) vs control, no explicit confound control, results dependent on single EEG device, no description of how TDA features are generated per fold, no mention of random seed control or variance estimation, no ablation studies
Ortiz et al., 2016	**NR**	YES	YES	subject-wise	YES	NR	NO	MOD	LOW	No preprocessing pipeline described, discussion of confounders and external validation missing, high-dimensional feature space from DBN may pose overfitting risk despite cross-validation, voxel selection or ROI preprocessing not specified.
Bloch et al., 2024	**YES**	YES	YES	subject-wise	YES	NR	YES	MOD	LOW TO MOD	Time-consuming preprocessing, DL models trained without class imbalance adjustments, transfer learning on artificial dataset not beneficial, excludes MCI and other relevant groups, no mention of scanner/site harmonization.
Deatsch et al., 2022	**YES**	YES	YES	subject-wise	YES	YES (age, gender, scanner, site,	YES	MOD TO HIGH	LOW TO MOD	No data augmentation or scanner/site harmonization, threshold may require tuning in clinical use, exclusion of subjects based on biomarker availability, longitudinal data limited to less than 3 timepoints with 1-year gaps, clinical data limited to age and gender, external validation accuracy lower

						acquisition methods)				
Mahendran et al., 2022	**YES**	YES	YES	NR	NR	NR	NO	MOD	MOD	Small sample size (overfitting risk), no demographic metadata or covariate adjustment, no external dataset used, no code or pipeline sharing limits reproduction, data splitting method unspecified
Yoshida et al., 2023	**NO**	NR	NR	subject-wise	NR	PARTIAL (age included, others discussed but not controlled)	NO	LOW	HIGH	No cross-validation or hold-out strategy used, small sample size with potential class imbalance & no stratified sampling described, use of age as both feature and stratifier, unclear model cutoff selection, use of commercial software with black-box functionality limits its reproducibility.
Drage et al., 2022	**YES**	YES	YES	NR	NR	NR	NO	MOD	HIGH	MCI underrepresented, no cross-validation, interpolation of small matrices could introduce artifacts, single-site data limits generalizability, no demographic/clinical variables used, potential label noise (AD diagnosis not pathology-confirmed), no test set held out for final unbiased evaluation, unclear test-time decision process.
Srivishagan et al., 2022	**YES**	YES	YES	subject-wise	YES	YES (age, sex)	NO	HIGH	LOW	Data from multiple scanners/vendors, Grad-CAM limited by architecture, no test-time ensembling or uncertainty quantification, structured input issues unaddressed, no discussion of MCI or multiclass prediction.
Tsang et al., 2019	**YES**	YES	YES	subject-wise	NR	NR	NO	MOD	MOD	Uses complex custom methods, no discussion of class imbalance, demographics not analyzed as confounders, external generalizability limited to Welsh population, interpretability limited beyond top 10 features, SAIL dataset access restricted, no model calibration assessment.
Bit et al., 2021	**YES**	YES	YES	subject-wise	YES	NR	YES	MOD	LOW	Small number of autopsy-confirmed AD cases, no confounder adjustment, external cohort relied only on CDR for diagnosis, preprocessing relies on standard FSL tools but harmonization across scanner/site not clearly detailed, codes not publicly available.
Rutkowski et al., 2022	**YES**	YES	YES	subject-wise	YES	NR	NO	MOD	LOW	Small/unbalanced sample (only 1 male), very high results suggest possible overfitting, data not publicly available, data augmentation via SMOTE used but not deeply analyzed or compared against other balancing methods.
Ozkaraca et al., 2021	**YES**	YES	YES	NR	YES	NR	NO	MOD	LOW	Lack of specificity on preprocessing details , dataset details under-described, high reported accuracy may be inflated without clear leakage prevention, no external dataset tested, no confounder analysis or demographic description.
Cardenas-Peña et al., 2021	**YES**	YES	YES	NR	NR	NO	NO	MOD	MOD	Risk of subject overlap is unclear due to lack of detail on data split; no covariate control; small sample size could inflate performance metrics.

Amezquita-Sanchez et al., 2021	**YES**	YES	YES	NR	NR	NO	NO	MOD	MOD	EEG segments from multiple subjects used, but unclear if subject-wise separation was maintained; no mention of confounder control or stratification.
Zaman et al., 2022	**YES**	YES	YES	NR	NR	NO	NO	MOD	MOD	Model trained on 2D MRI slices; splitting not clearly described, creating potential leakage risk. No demographic control.
Neira-Rodado et al., 2023	**YES**	YES	YES	NR	NR	NO	NO	MOD	MOD	Strong use of EEG epochs; potential leakage if segments from same subjects cross validation folds; no demographic adjustments mentioned.
Alarjani et al., 2023	**YES**	YES	YES	NR	NO	NO	NO	MOD	HIGH	Multiple CNNs compared; feature-level input from CAT12, but unclear data partitioning raises leakage risk. No demographic adjustment.
Park et al., 2019	**YES**	NO	YES	subject-wise	YES	NO	YES	MOD	LOW	Key limitation: AI model is a black box — no architecture or parameters reported; lacks confounder control but tests generalizability across sites.
Kar et al., 2021	**YES**	YES	YES	NR	NO	NO	NO	MOD	HIGH	Potential data leakage; training on preprocessed slices without clear subject-wise separation. No mention of age/sex controls.
Gong et al., 2021	**YES**	YES	YES	NR	NO	NO	NO	MOD	HIGH	Serious risk of slice-wise data leakage; uses 2D slices from 3D MRI without confirming subject-level separation; no mention of demographic control.
Gyawali et al., 2022	**YES**	YES	YES	NR	NO	NO	NO	MOD	HIGH	Risk of data leakage due to slice-based 2D model; subject-level grouping not discussed; no covariate adjustments.
Guelib et al., 2022	**YES**	YES	YES	subject-wise	Implicitly avoided	NR	NO	HIGH	LOW	NONE
Sethuraman et al., 2023	**yes**	YES	YES	NR	NR	NO	NO	MOD	MOD	No clarity on whether subject-wise splitting was used; no confounder adjustment; external validation absent.

Fristed et al., 2021	**YES**	YES	YES	subject-wise	YES	Yes (age, sex, scanner type controlled in analyses)	YES	HIGH	LOW	NONE
Chang et al., 2023	**YES**	YES	YES	NR	NR	NO	NO	MOD	MOD	Fusion strategy well-described, but unclear on whether cross-validation was subject-wise; no confounder control or external dataset.
Zhang et al., 2021	**YES**	YES	YES	NR	NR	NO	NO	MOD	MOD	Attention mechanism used for interpretability; possible risk of data leakage due to unclear splitting details; no external dataset or demographic adjustment discussed.
Basheera & Ram, 2021	**YES**	YES	YES	subject-wise	YES	NR	YES (OA-SIS)	HIGH	LOW	Clear preprocessing (entropy-based slice selection, skull stripping, segmentation), but lacks detail on demographic covariate handling.
Akhtar et al., 2022	**YES**	YES	YES	subject-wise	YES	Yes (age, sex, scanner)	NO	HIGH	LOW	Used multiple interpretable models including attention weights and SHAP; robust methodology with confounder consideration.
Wen et al., 2020	**YES**	YES	YES	subject-wise	YES	Yes (site, age, sex)	NO	HIGH	LOW	Clear best-practice approach, addressed confounding, leakage, and reporting very thoroughly. Strong benchmark study.
Choi et al., 2022	**YES**	YES	YES	subject-wise	YES	NR	NO	HIGH	LOW	Novel context-aware transformer network; ablation studies performed; no mention of covariate adjustment.
Gallucci et al., 2023	**YES**	YES	YES	subject-wise	YES	NR	NO	MOD	LOW	Used Grad-CAM for interpretability; applied ResNet variants; unclear if variables like age/sex were adjusted for; lacks mention of confounder impact.
Dong et al., 2020	**YES**	YES	YES	subject-wise	YES	NR	NO	HIGH	LOW	Multi-head attention + graph convolutional networks used for classification; detailed experimental setup; no mention of confounder control.
Jo et al., 2020	**YES**	YES	YES	subject-wise	YES	NR	NO	HIGH	LOW	Clear multimodal setup with fMRI and sMRI; fusion architecture explained well; interpretability approach outlined; no mention of demographic covariates.
Klingenberg et al., 2020	**YE**	YE	YE	subject-wise	YES	Yes (age, sex, MMSE,	YES	HIGH	LOW	Among the most rigorous: external validation across 4 datasets, confounder-adjusted models, SHAP for interpretability. Strong adherence to best practices.

	**S**	S	S			scanner, and more)				
Bi et al., 2020	**YES**	YES	YES	subject-wise	YES	NR	NO	HIGH	LOW	Uses dual-branch modality-specific networks and interpretable Grad-CAM attention maps; confounders like age or sex not discussed.
Luo et al., 2022	**YES**	YES	YES	subject-wise	YES	NR	NO	HIGH	LOW	Multimodal attention-based fusion is clearly described; interpretation techniques applied; no mention of controlling for confounders like age or sex.
Han et al., 2021	**YES**	YES	YES	subject-wise	YES	NR	NO	HIGH	LOW	Clear use of 3D-CAM for interpretability; solid internal validation; does not address confounder control (e.g., age, scanner variability).
Irie et al., 2020	**YES**	YES	YES	subject-wise	YES	NR	NO	HIGH	LOW	Small sample size mitigated with residual extraction approach; model compared to radiologists; lacks confounder handling; interpretability via Grad-CAM.
Ntracha et al., 2022	**YES**	YES	YES	subject-wise	YES	NR	NO	HIGH	LOW	Strong interpretability focus (Grad-CAM); internal validation only; lacks details on potential confounder adjustment (e.g., scanner, sex).
Song et al., 2021	**YES**	YES	YES	subject-wise	YES	NR	NO	HIGH	LOW	Strong focus on interpretability via Grad-CAM; subject-wise CV confirmed; minor omission in reporting of confounder handling (e.g., age, sex).
Xing et al., 2021	**YES**	YES	YES	Subject-wise	YES	NR	NO	MOD	LOW	Innovative Learnable Weighted Pooling approach; no external test set used; efficiency-focused but slightly less emphasis on biological interpretability.

Abbreviations: CV: Cross-Validation; LOOCV: Leave-One-Out Cross-Validation; NR: Not Reported; MOD: Moderate; sMRI: structural Magnetic Resonance Imaging; fMRI: functional Magnetic Resonance Imaging; DTI: Diffusion Tensor Imaging; PET: Positron Emission Tomography; EEG: Electroencephalography; Grad-CAM: Gradient-weighted Class Activation Mapping; SHAP: SHapley Additive exPlanations; SMOTE: Synthetic Minority Oversampling Technique; TDA: Topological Data Analysis; MoCA: Montreal Cognitive Assessment; CDR: Clinical Dementia Rating; MMSE: Mini-Mental

State Examination; DBN: Deep Belief Network; ROI: Region of Interest; FSL: FMRIB Software Library; CAT12: Computational Anatomy Toolbox; SAIL: Secure Anonymised Information Linkage.

Figure S2. Methodological Quality Heatmap Across Included Studies

Compliant/Low Risk | Partial/Moderate Risk | Non-compliant/High Risk | Not Reported

Study (Year)	Subject-wise Split	External Validation	Confounder Control	Sample Size ≥300	Clear Methods	Code Available	Multi-site Data	Cross-validation	Performance <90%	Overall Risk
Klingenberg et al. 2020	✓	✓	✓	✓	✓	✓	✓	✓	✓	LOW
Wen et al. 2020	✓	✗	✓	✓	✓	✓	✓	✓	✓	LOW
Fristed et al. 2021	✓	✓	✓	✗	✓	✓	✓	✗	✓	LOW
Yagis et al. 2021	✓	✗	✓	✗	✓	✗	✓	✓	✓	LOW
Deatsch et al. 2022	✓	✓	✓	✓	✓	✗	✓	✗	✓	LOW
Ortiz et al. 2016	✓	✗	NR	✓	?	✗	✓	✓	✓	LOW
Rutkowski et al. 2023	✓	✗	NR	✗	✓	✗	✗	✓	✗	LOW
Srivishagan et al. 2022	✓	✗	✓	✓	✓	✗	✓	✓	✓	LOW
Bit et al. 2021	✓	✓	NR	✓	?	✗	✓	✓	✓	LOW
Park et al. 2019	✓	✓	✗	✓	?	✗	✓	✓	✓	LOW
Basheera & Ram 2021	✓	✓	NR	✓	✓	✗	✓	✓	✓	LOW
Akhtar et al. 2022	✓	✗	✓	✗	✓	✓	✓	✓	✓	LOW
Choi et al. 2022	✓	✗	NR	✓	✓	✗	✓	✗	✓	LOW
Jo et al. 2020	✓	✗	NR	?	✓	✗	✓	✓	✓	LOW
Dong et al. 2020	✓	✗	NR	✓	✓	✗	✓	✓	✓	LOW
Bi et al. 2020	✓	✗	NR	✗	✓	✗	✓	✓	✓	LOW
Luo et al. 2022	✓	✗	NR	✓	✓	✗	✓	✓	✓	LOW
Han et al. 2021	✓	✗	NR	✓	✓	✗	✓	✓	✓	LOW
MODERATE RISK STUDIES										
Mahendran et al. 2022	NR	✗	NR	✓	?	✗	✓	✓	✗	MOD
Tsang et al. 2019	?	✗	NR	✓	?	✗	✗	✓	✗	MOD
Cardenas-Peña 2021	NR	✗	✗	✓	?	✗	✓	✓	?	MOD
Amezquita-S. 2021	NR	✗	✗	✗	?	✗	✗	✓	?	MOD
Zaman et al. 2022	NR	✗	✗	✓	?	✗	✓	✓	?	MOD
Neira-Rodado 2023	NR	✗	✗	✓	?	✗	✗	✓	?	MOD
Sethuraman 2023	NR	✗	✗	✓	?	✗	✓	✓	?	MOD
Chang et al. 2023	NR	✗	✗	✗	?	✗	✓	✓	?	MOD
Zhang et al. 2021	NR	✗	NR	✓	?	✗	✓	✓	?	MOD
HIGH RISK STUDIES										
Yoshida et al. 2023	✗	✗	Part.	✗	✗	✗	✗	✗	✗	HIGH
Drage et al. 2022	✗	✗	✗	✗	?	✗	✗	✗	✗	HIGH
Alarjani et al. 2023	✗	✗	✗	✓	?	✗	✓	✓	✗	HIGH
Kar et al. 2021	✗	✗	✗	✓	?	✗	✓	✓	✗	HIGH
Gong et al. 2021	✗	✗	✗	✓	?	✗	✓	✓	✗	HIGH
Gyawali et al. 2022	✗	✗	✗	✓	?	✗	✓	✓	✗	HIGH
OVERALL (n=44)	61.4%	15.9%	18.2%	68.2%	65.9%	22.7%	77.3%	84.1%	70.5%	61/25/14

Supplementary Table S3. Benchmark Articles Used to Validate Search Strategy

#	Reference	Contribution / Rationale
1	Litjens G, et al. (2017). *A survey on deep learning in medical image analysis.* Medical Image Analysis, 42, 60–88.	Widely cited survey establishing DL applications in medical imaging; foundational reference to ensure general methodological breadth.
2	Wen J, et al. (2020). *Convolutional neural networks for classification of Alzheimer's disease: reproducibility and evaluation.* Nature Communications, 11, 1952.	Benchmarking study on ADNI with explicit focus on reproducibility; essential for search validation in AD imaging.
3	Payan A, Montana G. (2015). *Predicting Alzheimer's disease: a neuroimaging study with 3D convolutional neural networks.* Frontiers in Neuroscience, 9, 220.	Among the earliest applications of CNNs to MRI for AD classification; serves as a methodological pioneer.
4	Vieira S, et al. (2017). *Using machine learning and structural MRI to predict dementia progression: A systematic review.* NeuroImage: Clinical, 16, 659–675.	Systematic review capturing early methodological trends in ML/DL for AD; ensures coverage of broader ML-to-DL transition.
5	Bron EE, et al. (2015). *Standardized evaluation of algorithms for computer-aided diagnosis of dementia based on structural MRI: the CADDementia challenge.* Neurobiology of Aging, 36, S153–S163.	Multi-center benchmarking challenge highlighting risks of overfitting; gold-standard reference for evaluating methodology.
6	Rieke N, et al. (2020). *The future of digital health with federated learning.* Nature Machine Intelligence, 2, 349–360.	Discusses reproducibility, privacy, and interpretability in DL for medical imaging; relevant to methodological safeguards.
7	Bae J, et al. (2020). *Interpretable deep learning for Alzheimer's disease classification using MRI.* Alzheimer's & Dementia: Diagnosis, Assessment & Disease Monitoring, 12, e12044.	Demonstrates interpretable CNN methods applied to AD neuroimaging; key reference for explainability.
8	Jo T, et al. (2019). *Deep learning in Alzheimer's disease: classification and feature relevance with multimodal data.* Frontiers in Aging Neuroscience, 11, 220.	Multimodal deep learning with ADNI; interpretable features; highlights integration of clinical + imaging data.
9	Choi H, Jin KH. (2020). *Predicting cognitive decline with deep learning of brain metabolism and amyloid imaging.* Scientific Reports, 10, 2203.	PET-based interpretable deep learning study; demonstrates modality diversity and interpretability focus.
10	Eitel F, et al. (2021). *Uncovering convolutional neural network decisions for diagnosing Alzheimer's disease on structural MRI: a layer-wise relevance propagation study.* Human Brain Mapping, 42, 3453–3466.	Applies explainable AI (LRP) to AD classification, linking DL outputs to clinically meaningful features.
